# Large-Scale Biomonitoring of Remote and Threatened Ecosystems via High-Throughput Sequencing

**DOI:** 10.1371/journal.pone.0138432

**Published:** 2015-10-21

**Authors:** Joel F. Gibson, Shadi Shokralla, Colin Curry, Donald J. Baird, Wendy A. Monk, Ian King, Mehrdad Hajibabaei

**Affiliations:** 1 Biodiversity Institute of Ontario and Department of Integrative Biology, University of Guelph, Guelph, Ontario, Canada; 2 Environment Canada, Canada Centre for Inland Waters, Burlington, Ontario, Canada; 3 Environment Canada, Canadian Rivers Institute, University of New Brunswick, Fredericton, New Brunswick, Canada; 4 Canadian Rivers Institute, University of New Brunswick, Fredericton, New Brunswick, Canada; Consiglio Nazionale delle Ricerche (CNR), ITALY

## Abstract

Biodiversity metrics are critical for assessment and monitoring of ecosystems threatened by anthropogenic stressors. Existing sorting and identification methods are too expensive and labour-intensive to be scaled up to meet management needs. Alternately, a high-throughput DNA sequencing approach could be used to determine biodiversity metrics from bulk environmental samples collected as part of a large-scale biomonitoring program. Here we show that both morphological and DNA sequence-based analyses are suitable for recovery of individual taxonomic richness, estimation of proportional abundance, and calculation of biodiversity metrics using a set of 24 benthic samples collected in the Peace-Athabasca Delta region of Canada. The high-throughput sequencing approach was able to recover all metrics with a higher degree of taxonomic resolution than morphological analysis. The reduced cost and increased capacity of DNA sequence-based approaches will finally allow environmental monitoring programs to operate at the geographical and temporal scale required by industrial and regulatory end-users.

## Introduction

Many ecological studies rely on measuring biodiversity within each sample (alpha biodiversity), between sampling locations (beta diversity), and at the regional scale (gamma diversity). Incidence- and abundance-based metrics like taxon richness, taxon evenness, sample dissimilarity, and assemblage variation can all be derived from direct measures of biodiversity within environmental samples. Using biodiversity metrics, the temporal and spatial drivers of ecosystems can be explored [1–3]. Also, maintenance of baseline alpha, beta, and/or gamma diversity corresponds with increased ecosystem functionality and provision of ecosystem services [4]. In order to determine the impact of anthropogenic and natural stressors on aquatic ecosystems, it is first necessary to establish baseline biodiversity metrics for communities in a variety of regions. The lack of such baseline data has made it difficult to determine the nature and magnitude of biodiversity changes in response to stressors [5].

The Athabasca and Peace Rivers, together with the smaller Birch River, form a confluence in the Peace-Athabasca delta, the largest inland freshwater delta complex in the world, a UNESCO World Heritage Site (designated 1983), and a Ramsar Convention wetland of international importance (designated 1982). The entire Peace-Athabasca delta is contained within Wood Buffalo National Park, the largest national park in Canada, and second largest in the world. Of particular concern is the proximity of the Peace-Athabasca delta to expanding energy extraction (Alberta Oil Sands) and hydroelectric projects. The need for ecological assessments of these important boreal regions for early detection and management of potential anthropogenic effects is critical [5].

The goal of biomonitoring programs has been to assess human impacts against a background of natural variability in ecosystem structure, and biodiversity metrics have played a key role in achieving this [6]. For example, Environment Canada’s Canadian Aquatic Biomonitoring Network (CABIN) and the Australian River Assessment System (AUSRIVAS) employ standardized methods to collect and analyze environmental samples in order to assess freshwater site conditions and determine conservation and remediation actions. While varying widely in the exact protocols employed, existing efforts rely almost exclusively on sorting and morphological identification of collected benthic invertebrate specimens [6]. Such identification methods require a large investment of time, expertise, and money. These issues have greatly limited biomonitoring programs in remote sites, especially in ecosystems adjacent to large-scale energy and mining operations in less populated regions (e.g., Alberta Oil Sands, Canada). In order to maximize efficiency, identifications are often only conducted to higher taxonomic levels (e.g., family or order [7]).

As an alternative to morphological identification, the use of DNA sequence-based identification has been proposed [8]. The mitochondrial cytochrome *c* oxidase subunit I (COI) region has been demonstrated to be effective for the identification of Metazoa and is represented by hundreds of thousands of curated sequences in online sequence databases [9,10]. In direct comparisons, the use of COI sequence data has increased the taxon richness estimates of benthic samples by as much as 50% over expert identifications and 400% over amateur identifications [11]. Furthermore, direct tests of morphological identification versus DNA barcoding of individual benthic specimens indicate that DNA sequence data provide increased statistical power for bioassessment, mostly due to the addition of genus- and species-level identifications [12–15].

The conventional, Sanger method of DNA sequencing is capable of only producing single DNA sequences from individual specimens. High-throughput sequencing (HTS) employs parallel sequencing to generate millions of DNA sequences simultaneously. The HTS approach has been employed to recover DNA sequence data from constructed or pre-sorted mixtures of organisms [16–18]. Detailed species-, genus-, family-, and order-level taxonomic information has also been successfully recovered from unsorted mixtures of metazoan tissue collected via freshwater benthic samples and terrestrial Malaise traps [19,20].

Until now, no-one has determined whether an HTS approach can be used to determine alpha, beta, and gamma diversity metrics for freshwater benthic samples collected as a part of a large-scale biomonitoring program in remote sites. Moreover, little evidence has been provided to support the assertion that the added taxonomic resolution supplied by HTS methods will provide a more complete snapshot of site- and region-level biodiversity metrics. It is proposed that HTS-generated taxonomic data will provide higher levels of discrimination between samples, sites, and ecosystems than that provided by conventional morphological analysis. Furthermore, it is hypothesized that HTS-generated measures of biodiversity resolved at increasing levels of taxonomic detail (e.g., genus versus order level) will provide similar patterns between samples, but with added statistical power.

## Methods

### Field sampling

Field permits were granted by Parks Canada at Wood Buffalo National Park and samplings were conducted by Environment Canada and Parks Canada staff. The field work did not involve endangered or protected species. Four riverine wetland sampling sites from each of the Peace and Athabasca deltas were sampled in June, 2012. Full collection site data is found in Table A in S1 File. Three replicate samples of the aquatic invertebrate assemblage were taken from the edge of the emergent vegetation zone into the submerged vegetation zone at each site. Replicates were located approximately 50 metres apart. Samples were collected using a standard Canadian Aquatic Biomonitoring Network (CABIN) pond net with a sterile 400μm mesh net and attached collecting cup attached to a pole. Effort was standardized at two minutes per sample. Sampling was conducted by moving the net up and down through the vegetation in a sinusoidal pattern while maintaining constant forward motion. If the net became impeded by dislodged vegetation, sampling was paused so extraneous vegetation could be removed. Sampling typically resulted in a large amount of vegetation within the net. After sampling, this vegetation was vigorously rinsed to dislodge attached organisms and visually inspected to remove remaining individuals before discarding. The remaining material was removed from the net and placed in a sterile, white 1L polyethylene sample jar filled no more than half full. The net and collecting cup were rinsed and inspected to remove any remaining invertebrates. Samples were preserved in 95% ethanol in the field, and placed on ice in a cooler for transport to the field base. Here they were transferred to a freezer before shipment. A clean net was used to collect samples at each site and field crew wore clean nitrile gloves to collect and handle samples in the field and laboratory, thereby minimizing the risk of DNA contamination between sites.

### Morphological identification

All samples were then submitted to morphological analysis in accordance with CABIN protocol (heretofore referred to as CABIN). Samples were immediately placed in a -80˚C freezer upon receipt in the lab. Full procedures regarding morphological processing and identification are outlined in the CABIN laboratory manual [7]. Briefly, replicate samples were subsampled using a 100-cell Marchant box [21]. The cells to be identified are randomly selected in advance. Successive cells are processed completely until at least 300 individuals have been identified, with a minimum of five cells being processed. Total abundances are then extrapolated based on number of cells processed. Identification was conducted to family level, although for some groups only class- or order-level identification is recovered. Morphological abundance lists were generated based on mixed taxonomic levels. The morphological data is publicly available from Canada-Alberta Oil Sands Environmental Monitoring Information Portal (www.jointoilsandsmonitoring.ca). After sorting and identification, the samples were reconstituted and preserved again in 95% ethanol.

### DNA extraction

Each of the benthic samples was homogenized in 95% ethanol and the resultant slurry was transferred to multiple 50mL Falcon tubes. After ethanol evaporation of the slurry at 56°C, the dried mixture was divided into three lysing matrix tubes “A” (about 100 mg each) and further homogenized using an MP FastPrep-24 Instrument (MP Biomedicals Inc.) at speed 6 for 40 sec. Total DNA of this homogenized slurry was extracted using a Nucleospin tissue kit (Macherey-Nagel Inc.) following the manufacturer’s instructions and eluted in 50μL of molecular biology grade water.

### PCR amplification and high-throughput sequencing

Two fragments within the standard COI DNA barcode region were amplified with two primer sets in a two-step PCR amplification regime [19]. The F230 fragment of COI is approximately 230bp in length, is found at the 5’ end of the standard barcoding region and is amplified using the standard Folmer et al. forward primer (F GGTCAACAAATCATAAAGATATTGG [22]) and a reverse primer newly designed for this study using sequences from a wide range of arthropod orders (230_R CTTATRTTRTTTATICGIGGRAAIGC). The BE fragment of COI is approximately 314bp in length, is found toward the 3’ end of the standard barcoding region, and has no overlap with the F230 region. The BE fragment id amplified using the following primers, previously optimized for use with a broad range of arthropod orders: B CCIGAYATRGCITTYCCICG [19], and R5 GTRATIGCICCIGCIARIAC [20]. The first PCR used COI specific primers and the second PCR involved Illumina-tailed primers. The PCR reactions were assembled in 25μL volumes. Each reaction contained 2μL DNA template, 17.5μL molecular biology grade water, 2.5μL 10× reaction buffer (200mM Tris HCl, 500mM KCl, pH 8.4), 1μL MgCl_2_ (50mM), 0.5μL dNTPs mix (10mM), 0.5μL forward primer (10mM), 0.5μL reverse primer (10mM), and 0.5μL Invitrogen’s Platinum Taq polymerase (5 U/μL). The PCR conditions were initiated with heated lid at 95°C for 5min, followed by a total of 30 cycles of 94°C for 40s, 46°C (for both primer sets) for 1min, and 72°C for 30s, and a final extension at 72°C for 5min, and hold at 4°C. Amplicons from each sample were purified using Qiagen’s MiniElute PCR purification columns and eluted in 30μL molecular biology grade water. The purified amplicons from the first PCR were used as templates in the second PCR with the same amplification condition used in the first PCR with the exception of using Illumina-tailed primers in a 30-cycle amplification regime. All PCRs were done using Eppendorf Mastercycler ep gradient S thermalcyclers and negative control reactions (no DNA template) were included in all experiments. PCR products were visualized on a 1.5% agarose gel to check the amplification success. All generated 48 amplicons plates were dual indexed and pooled into a single tube and sequenced on a Miseq flowcell using a V2 Miseq sequencing kit (250 × 2)(FC-131-1002 and MS-102-2003). All sequencing data generated has been submitted to Dryad and can be accessed at doi:10.5061/dryad.vm72v.

### Bioinformatic processing

For all 24 samples, a total of 11.64 million Illumina reads were generated from both COI fragments. For each sample, the forward and reverse raw reads for the BE fragment and the F230 fragment were merged with SEQPREP software (https://github.com/jstjohn/SeqPrep) requiring a minimum overlap of 25bp and no mismatches, resulting in 5.8 million total paired-end reads (mean—261,930 reads/sample). All Illumina paired-end reads were filtered for quality using PRINSEQ software [23] with a minimum Phred score of 20, window of 10, step of 5, and a minimum length of 100bp. A total of 1.02 million paired BE reads (mean—59,820 reads/sample) and a total of 4.37 million paired F230 reads (mean—182,120 reads/sample) were retained for further processing. USEARCH v6.0.307 [24] with the UCLUST algorithm was used to de-replicate and cluster the remaining sequences using a 99% sequence similarity cutoff. This was done to denoise any potential sequencing errors prior to further processing. Chimera filtering was performed using USEARCH with the ‘de novo UCHIME’ algorithm [25]. At each step, cluster sizes were retained, singletons were retained, and only putatively non-chimeric reads were retained for further processing. All filtered, non-chimeric reads from all 24 samples were pooled and clustered at 98% similarity using USEARCH. For those clusters including at least 100 sequences, membership in each cluster for each sample was recorded as an OTU sequence abundance matrix (DNA-OTU).

Both BE and F230 sequences were pooled for each sample and identified using the MEGABLAST algorithm [26] against a reference library. This reference library contained all verified COI sequences downloaded from the GenBank database September 5^th^ 2014 with a minimum length of 100bp (N = 985,210 sequences). All MEGABLAST searches were conducted with a minimum alignment length percentage of 85% and a minimum similarity of 90%. Taxonomic identifications were recovered based on unambiguous top matches. Genus, family, and order matrices for taxa with a minimum of ten sequences per sample were generated for each sample based on these matches (heretofore referred to as DNA-order, DNA-family, DNA-genus). Only taxon names within benthic metazoan phyla (i.e., Annelida, Arthropoda, Mollusca, Chordata, Cnidaria) were included in analysis. A subset of matches with a minimum similarity of 98% was used to generate a species matrix (DNA-species). For all identification levels, except DNA-OTU, a subset of the matrix including only representatives of Ephemeroptera, Trichoptera, and Odonata (ETO) was generated.

### Biodiversity analysis

Species, genus, family, order, OTU, and mixed taxonomic level morphological matrices (hereby designated DNA-species, DNA-genus, DNA-family, DNA-order, DNA-OTU, and CABIN, respectively) were used to calculate richness and evenness values for each sample. Beta diversity metrics were calculated for eight sites (three samples each), two rivers (twelve samples each), and all samples combined (24 samples). Similarity between means of biodiversity metrics between river sites was determined using a Welch’s t-test, which accounts for unequal variances between groups. All richness and dissimilarity metrics were calculated using the *vegan* 2.2–1 package [27] in R v3.1.2 [28]. We used a Pearson’s product-moment correlation test to determine correlation between alpha diversity metrics gathered through different methods and at different taxonomic levels.

We used average dissimilarity of samples from their group centroid in principal coordinate space to assess the assemblage variation [29, 30]. The presence/absence-based Sørensen dissimilarity index the abundance-based Bray-Curtis dissimilarity index based on raw observations (individuals for CABIN, sequences for DNA methods), and Bray-Curtis dissimilarity based on proportion of overall observations (individuals or sequences), were all used to analyze differences in assemblage variation using a distance-based test for homogeneity of multivariate dispersions. Analyses were completed using the *betadisper* function in *vegan*. Bray-Curtis dissimilarity values based on proportion of observations were also used to calculate a non-metric multi-dimensional scaling (nMDS) analysis using the *vegdist* and *metaMDS* commands in *vegan*.

## Results and Discussion

### Raw data

**Fig 1:**
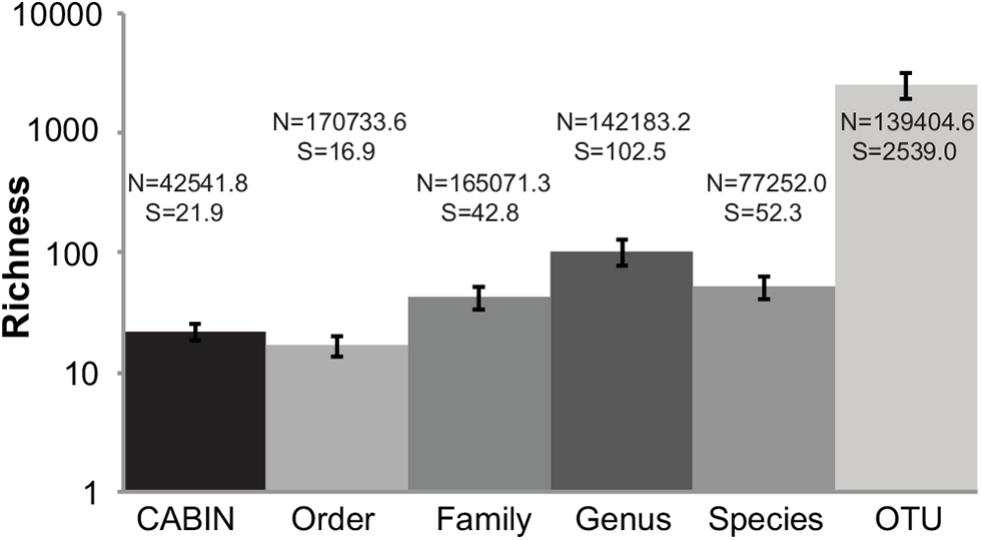
Raw biodiversity data for 24 benthic samples from the Peace-Athabasca delta at six different taxonomic levels via morphological identification and HTS. Mean taxon richness (S) of all samples plus one standard deviation is depicted. N denotes the mean number of sequences identified at this taxonomic level per sample.

The CABIN protocol involves subsampling benthic samples, identifying individuals to certain taxonomic level, and extrapolating to an estimated total abundance [7]. A mean of 2,030.67 (range: 839–7,841) individuals were identified morphologically per sample, producing an estimated mean total abundance of 42,541.81 (range: 17,100–155,600) individuals per sample (Fig 1). A mean of 262,126.6 (range: 169,199–357,609) total DNA sequences were recovered per sample by the Illumina MiSeq. Following quality filtering, a mean of 241,719.4 (range: 168,044–353,164) total good length, good quality DNA sequences were available. The number of sequences recovered through OTU clustering and identified at each taxonomic level varied from a mean of 77,252 (SD ± 37,224.68) sequences per sample at the species level up to 170,733 (± 56,179.01) sequences per sample at the order level (Fig 1).

### Alpha diversity metrics

Estimated taxonomic richness varied from a mean of 21.88 (± 3.67) per sample at the CABIN level up to a mean of 2,539.00 (± 607.27) per sample at the DNA-OTU level (Fig 1). A significant, positive correlation was detected between Simpson index values calculated at the DNA-order and DNA-family levels, DNA-order and DNA-genus levels, DNA-family and DNA-genus levels, and DNA-genus and DNA-species levels (Table 1). A significant, positive correlation was detected between Pielou’s evenness values calculated at the DNA-order and DNA-family levels, DNA-order and DNA-genus levels, DNA-family and DNA-genus levels, and DNA-genus and DNA-species levels (Table 1).

**Table 1:** Pearson product moment correlations between alpha diversity metrics calculated at different taxonomic levels via morphological identification and HTS.

	Simpson index	Pielou’s evenness	ETO richness	%ETO
**CABIN vs. DNA-order**	0.271	0.119	0.085	**0.738**
**CABIN vs. DNA-family**	0.243	0.124	**0.518**	**0.735**
**CABIN vs. DNA-genus**	-0.182	-0.097	**0.534**	**0.707**
**CABIN vs. DNA-species**	-0.195	-0.019	**0.664**	**0.645**
**CABIN vs. DNA-OTU**	0.347	0.136	N/A	N/A
**DNA-order vs. DNA-family**	**0.966**	**0.931**	0.291	**1.000**
**DNA-order vs. DNA-genus**	**0.565**	**0.614**	0.317	**0.985**
**DNA-order vs. DNA-species**	0.225	0.210	0.364	**0.919**
**DNA-order vs. DNA-OTU**	0.127	-0.118	N/A	N/A
**DNA-family vs. DNA-genus**	**0.599**	**0.717**	**0.893**	**0.717**
**DNA-family vs. DNA-species**	0.231	0.285	**0.765**	0.285
**DNA-family vs. DNA-OTU**	0.148	-0.168	N/A	N/A
**DNA-genus vs. DNA-species**	**0.826**	**0.755**	**0.803**	**0.755**
**DNA-genus vs. DNA-OTU**	0.117	-0.335	N/A	N/A
**DNA-species vs. DNA-OTU**	0.017	-0.210	N/A	N/A

Highlighted values indicate a significant (*p*<0.05), positive correlation between indices at two different taxonomic levels.

**Fig 2:**
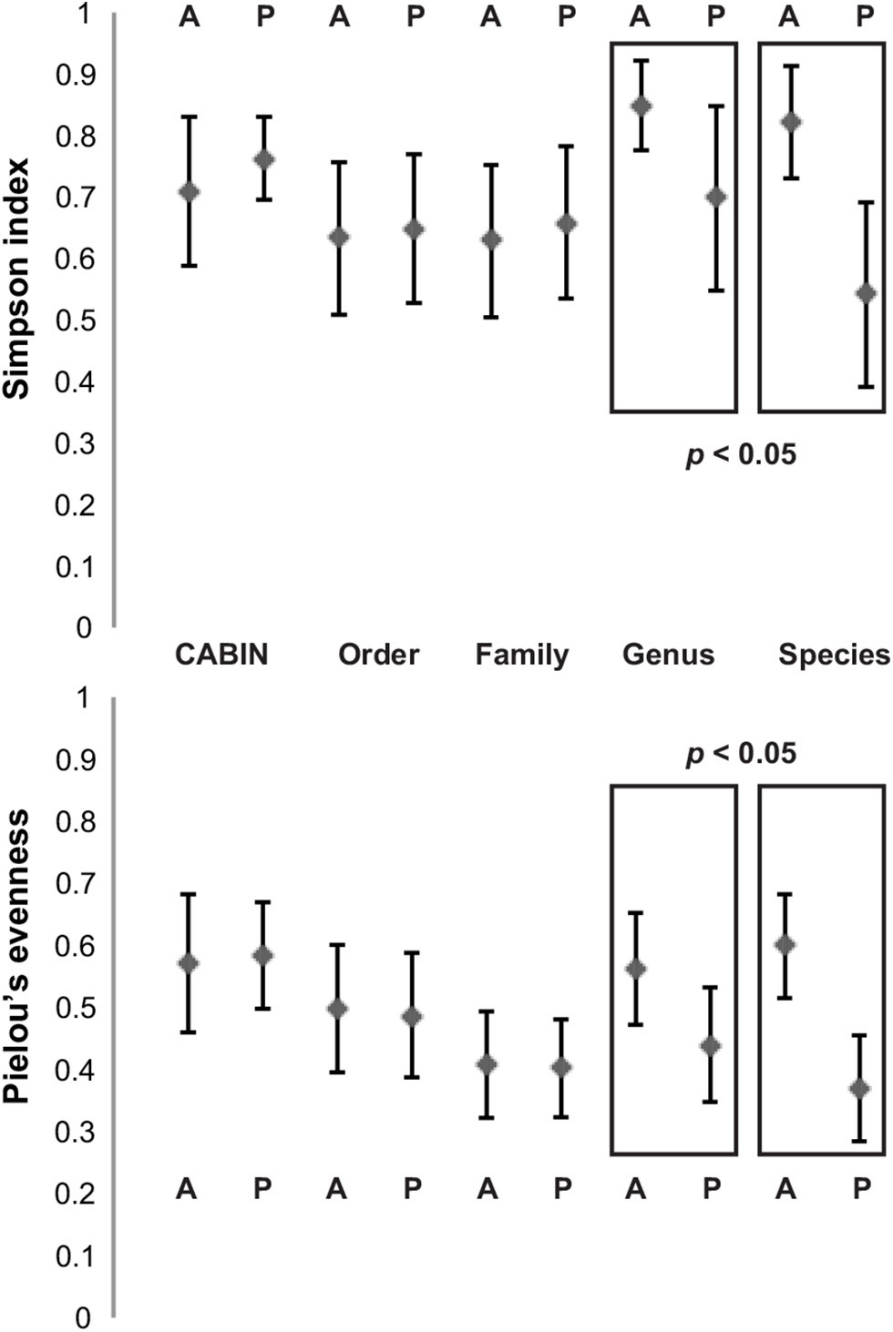
Alpha diversity metrics calculated for 24 benthic samples from the Peace-Athabasca delta at five different taxonomic levels via morphological identification and HTS. Means of all samples plus one standard deviation are depicted. Taxonomic levels at which a significant difference (*p*<0.05) between means of values for Athabasca and Peace sites are highlighted. A—Athabasca River sites. P–Peace River sites.

Mean Simpson index did not significantly differ between Athabasca wetland and Peace wetland sites when calculated at CABIN, DNA-order, or DNA-family levels (Fig 2; Table B in S1 File). However, when calculated at DNA-genus and DNA-species levels, a significant difference between the two sets of sites was detected (Welch two sample t-test–DNA-genus t = 3.12, df = 15.90, p = 0.007; DNA-species t = 5.46, df = 18.15, p < 0.001). Likewise, mean Pielou’s evenness did not significantly differ between Athabasca and Peace sites when calculated at CABIN, DNA-order, or DNA-family levels (Fig 2; Table B in S1 File). However, when calculated at DNA-genus and DNA-species levels, a significant difference between the two sets of sites was detected (Welch two sample t-test–DNA-genus t = 3.29, df = 21.97, p = 0.003; DNA-species t = 6.69, df = 22.00, p < 0.001). Additional alpha diversity metrics are included in Table B in S1 File.

Taxonomic richness varied between taxonomic levels in part due to differing levels of discrimination (Fig 1). Taxa recovered morphologically included a mix of classes, orders, and families. As such, the average richness reported is higher than when order richness, determined with DNA sequence data, is considered. Average family and genus richness, as determined with DNA sequence data, is higher than both morphological richness and DNA-order richness due to consistently increased discriminatory power at these levels. Average species richness, as determined with DNA sequence data, is lower compared to DNA-genus due to the stricter level of sequence similarity to published databases (i.e., 98% versus 90% sequence similarity) employed by our protocol. Average OTU richness is much higher than any other richness measure despite a comparable number of observations used (Fig 1). The greatly increased richness at this level is likely due to the inclusion of a large number of taxa not included at other levels, namely plants, algae, bacteria, and fungi. A degree of genetic differentiation, beyond the species level (i.e., population and haplotype differences) is also reported as increased OTU richness.

Derived alpha diversity metrics (i.e., Shannon index and Pielou’s evenness) show a similar pattern between samples when measured at DNA-order, DNA-family, DNA-genus, or DNA-species levels (Table 1). However, there is a lack of significant, positive correlation between these metrics derived from morphology or DNA-OTU as compared to any other level. This would suggest that alpha diversity could be consistently reported at a variety of taxonomic levels using DNA sequence data. Morphology, as implemented in the CABIN protocol, however, reports differing values due to an inability to consistently observe a targeted, morphologically identifiable subset of organisms [31]. Conversely, alpha diversity as derived from OTUs represents a much larger suite of included taxa and therefore different patterns. This lack of correlation between morphological and DNA-based alpha diversity metrics, especially when morphological inclusion is limited, has been previously noted [18].

When attempting to distinguish between wetland sites based on alpha diversity metrics, CABIN identification, order-level DNA sequence data, and family level DNA sequence data did not display any discriminatory resolution (Fig 2). It is only with the greater number of taxonomic units present in DNA-genus and DNA-species data that it is possible to observe differences between the river deltas in regards to Shannon index and Pielou’s evenness.

### Beta diversity metrics

Permutational ANOVA tests based on Sørensen, Bray-Curtis (sequence number), and Bray-Curtis (proportion of sequences) dissimilarities revealed that wetland and individual site were both significant sources of variation at all levels (CABIN, DNA-Order, DNA-Family, DNA-Genus, DNA-Species) (Table B in S1 File).

**Fig 3:**
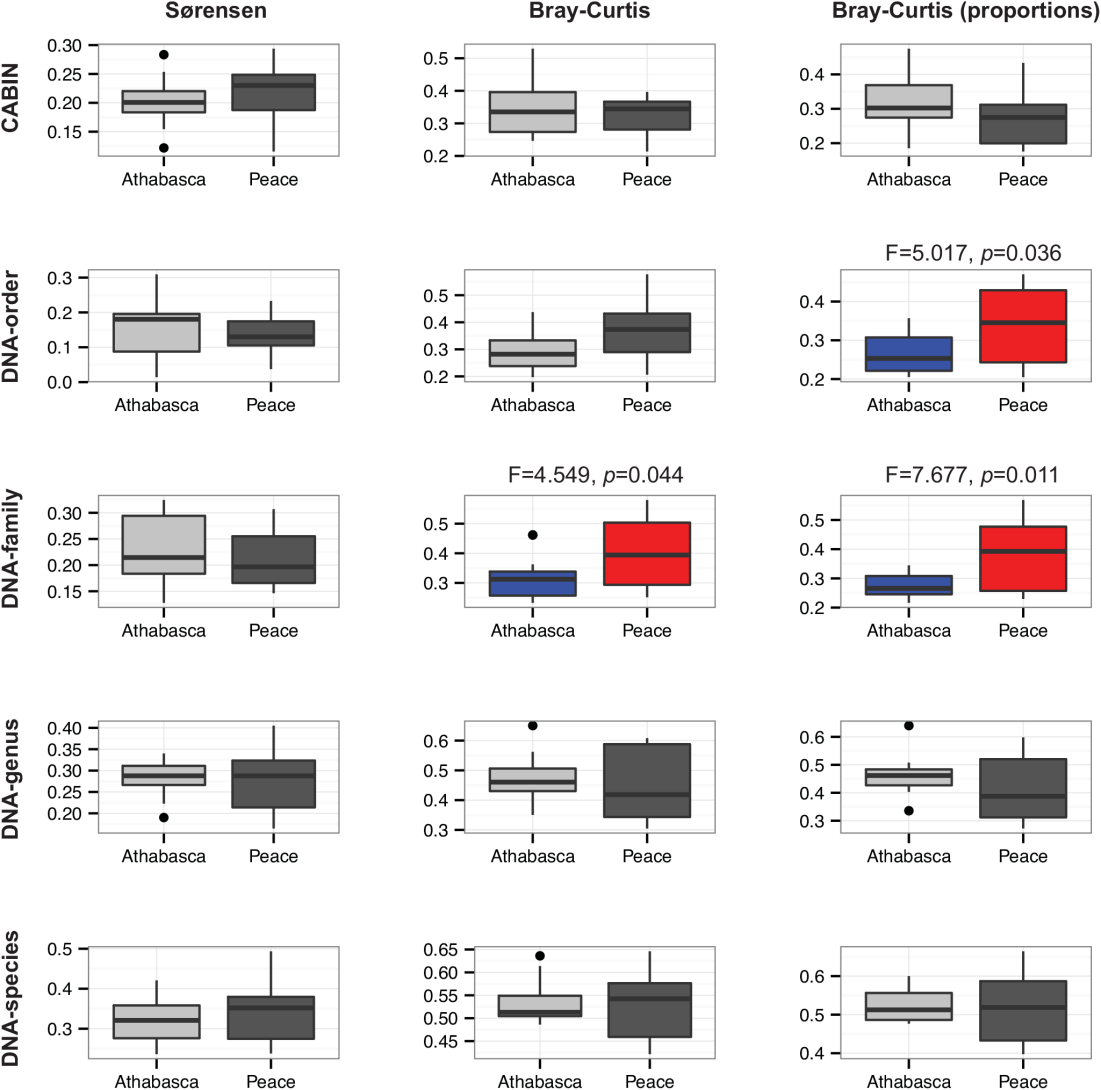
Community assemblage measures for 24 benthic samples from the Peace-Athabasca delta at five different taxonomic levels via morphological identification and HTS. Box and whisker plots of distance to centroid in principal coordinate space based on three dissimilarity measures–Sørensen, Bray-Curtis based on sequence number, and Bray-Curtis based on proportions of sequences. Significant differences (*p*<0.05) between Athabasca and Peace sites are highlighted in colour.

Beta diversity, as measured by assemblage variation using average dissimilarity of samples from their group centroid in principal coordinate space, could be calculated at all taxonomic levels. This approach to beta diversity analysis is positively related to environmental heterogeneity between groups of sites [30]. A greater degree of assemblage variation was recovered for the Peace River sites as compared to the Athabasca River sites (Fig 3). The detection of this difference was dependent on the taxonomic level and dissimilarity metric employed. The DNA-order and DNA-family levels recovered a significant beta diversity difference between river systems, whereas CABIN data did not. Once again, this is due to the increased discriminatory power afforded by DNA-based approaches. This ability to discriminate between groups of samples based on beta diversity measures using only HTS data has been demonstrated in the past, albeit not with aquatic samples and not at multiple levels of taxonomic discrimination [18].

Discriminatory power may have been lost at the genus and species level due to inherent limitations of the DNA amplification and sequencing process. PCR amplification primers can preferentially amplify some species DNA templates over others. This can lead to overrepresentation of some species and underrepresentation of others [32, 33]. However, PCR bias is not likely to have as much of an effect at higher taxonomic levels (family, order) as it does at the species or genus level [34].

**Fig 4:**
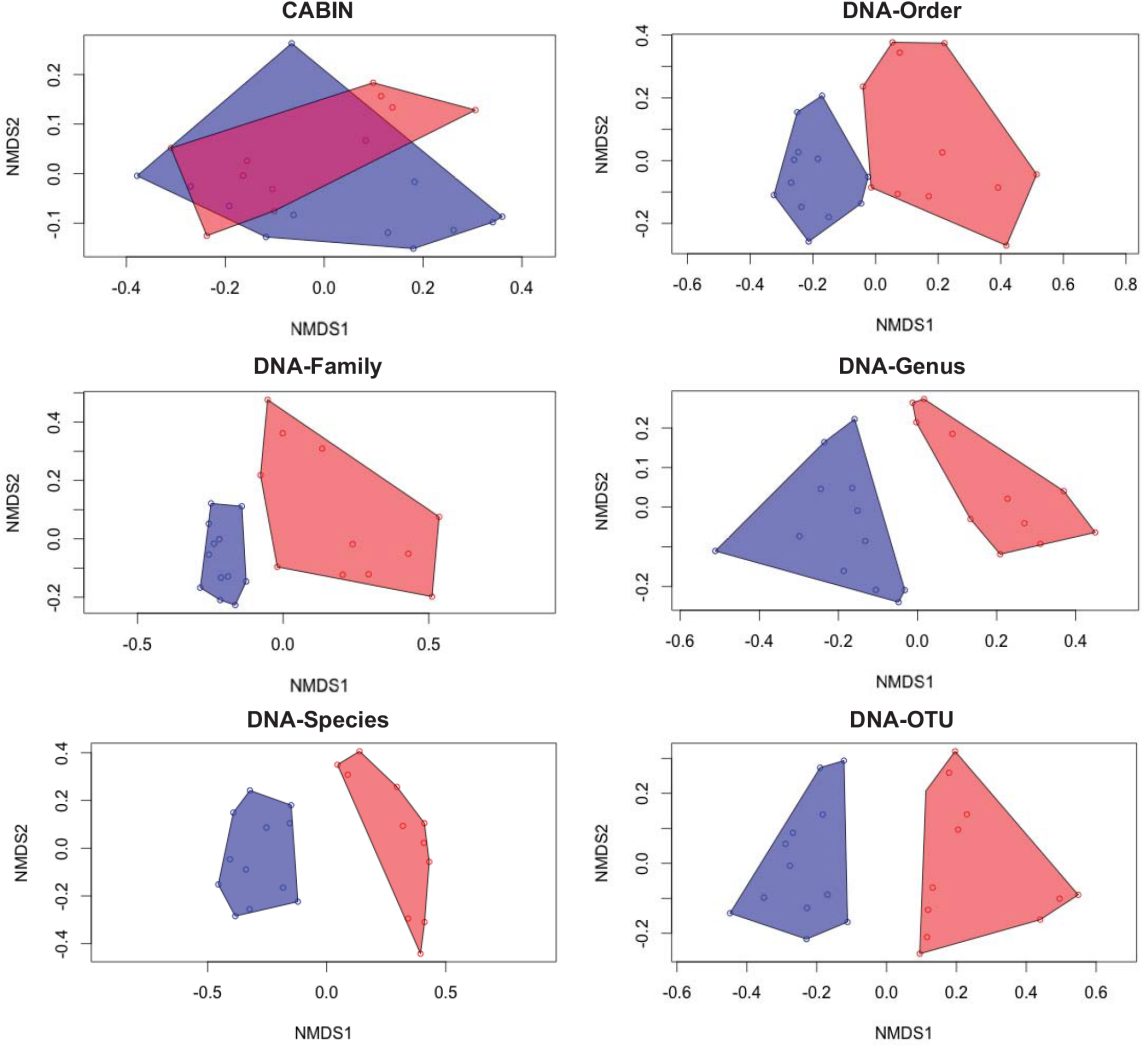
Community assemblage measures for 24 benthic samples from the Peace-Athabasca delta at six different taxonomic levels via morphological identification and HTS. Non-metric multidimensional scaling (nMDS) plots calculated from Bray-Curtis dissimilarities based on proportion of observations. Blue points and polygon represent Athabasca River sites. Red points and polygon represent Peace River sites.

Previous, morphology-based, aquatic biomonitoring studies have demonstrated the discriminatory power of abundance metrics [35, 36]. It has been claimed, however, that the complexities of sequence-based biodiversity measurement (e.g., PCR bias, gene copy number, individual biomass) will preclude the inclusion of abundance metrics [18]. The present study has sought to test this assumption. Incidence-based dissimilarity metrics (i.e., Sørensen) did not recover significant beta diversity differences whereas abundance-based metrics (i.e., Bray-Curtis) either including raw or proportional abundance did. An nMDS illustration of beta diversity echoes the discriminatory power of DNA-based metrics as only morphology-based identification failed to discriminate between wetland sites (Fig 4).

### Gamma diversity metrics

**Fig 5:**
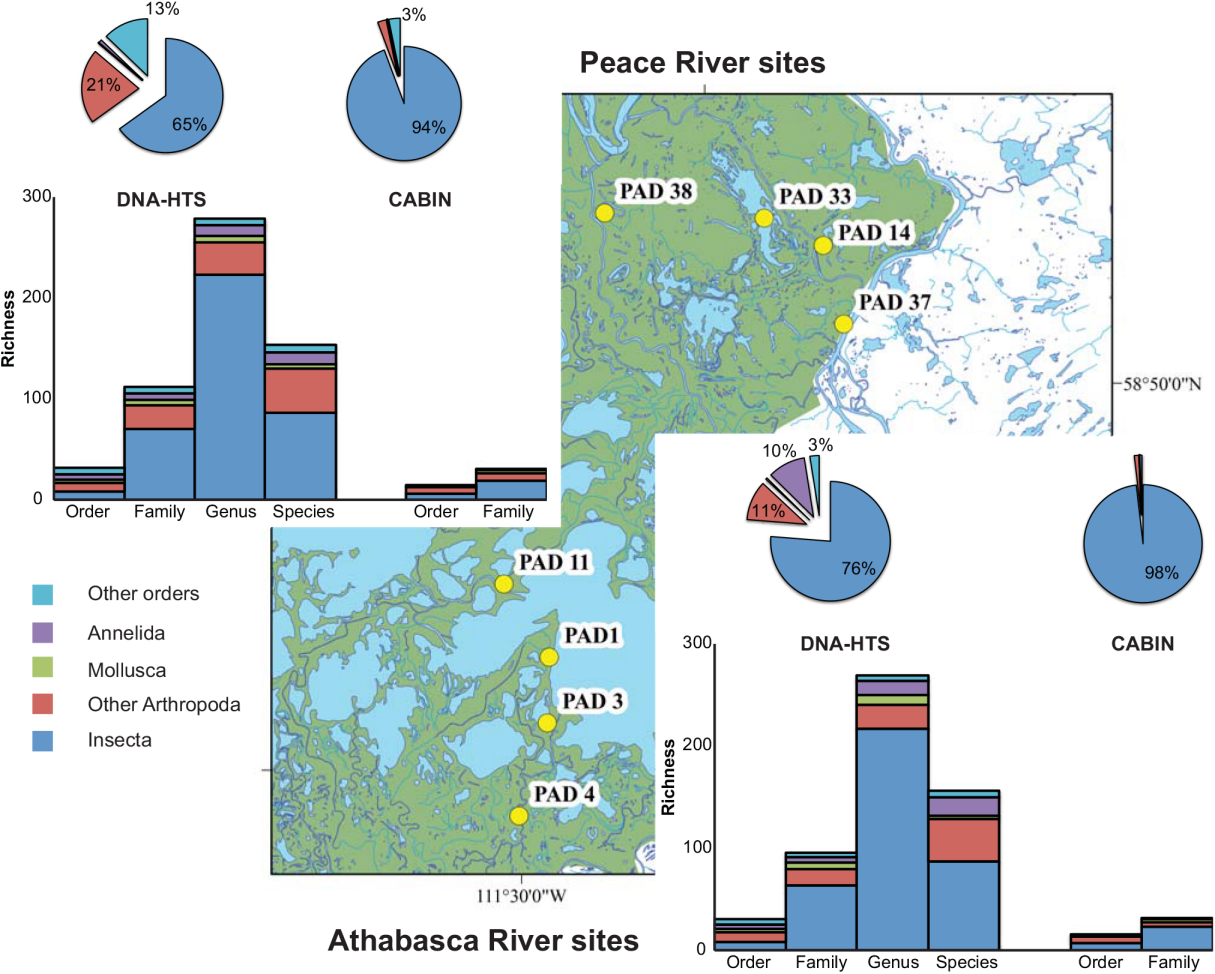
Gamma diversity recovered from Athabasca River and Peace River sites at six different taxonomic levels via morphological identification (CABIN) and molecular methods (DNA-HTS). Column charts represent taxonomic richness separated by major groups and taxonomic levels. Pie charts represent proportion of observations (sequences or individuals) identified to the order level assigned to each major group.

The number of taxa found in both Athabasca and Peace River wetland sites varied depending on taxonomic level (Fig 5). The proportion of taxa shared by both rivers ranged from 41.6% (DNA-species) to 63.2% (DNA-order). The proportion of taxa endemic to only one river system ranged from 15.7% (DNA-order Peace only) to 30.9% (DNA-family Athabasca only). CABIN recovered three order names and eleven family names not detected by HTS. While the level of morphological identification could have been increased with a greater investment of time and expertise, the adoption of a standardised CABIN protocol limits the diversity metrics to those recovered. Conversely, HTS recovered 22 order names and 108 family names not recovered by morphological identification. All 378 genus names and 219 species names were only recovered by HTS.

In the broadest sense, the total amount of biological information available regarding each of these river systems (i.e., gamma diversity) is greatly increased through the use of HTS technology. Over 700 family, genus, and species names are added to the reported biological richness by using DNA sequence-based identification as compared to morphological identification (Fig 5). Some of these names represent organisms present as DNA fragments (e.g., waste tissue, gut contents) and, therefore, not directly detectable using taxonomic keys. For example, the spottail shiner (*Notropis hudsonius* (Clinton)) and northern pike (*Esox Lucius* Linnaeus) were only detected in Athabasca sites. In other cases, DNA sequence identification affords greater levels of detail than family-level morphological identification. For example, three species of mayflies (*Caenis amica* Hagen, *C*. *latipennis* Banks, and *C*. *punctata* McDunnough) are found only in Peace sites.

This ability to recover genus and species names with a high degree of confidence is linked to the availability of reference DNA sequences for targeted taxonomic groups (i.e., North American arthropods). This same level of identification would not be likely if a group with a lower proportion of databased DNA barcode sequences was used (e.g., tropical arthropods [18,20]). Furthermore, the addition of family, genus, and species names allows the possibility of adding trait data, stressor tolerance, and trophic information to taxonomic lists [37–42].

### Targeted biodiversity metrics

Focusing biodiversity metrics on key “indicator” taxa has been a means of focusing biomonitoring efforts. Taxon-specific metrics (e.g., proportion of Ephemeroptera, Trichoptera, and Odonata—%ETO [41]) are used as proxies for overall biodiversity. Direct tests of such proxy approaches have revealed that taxon-limited biodiversity metrics may not accurately reflect general biodiversity patterns [11, 43–45].

Genus level biodiversity metrics of targeted benthic macroinvertebrates (Ephemeroptera, Plecoptera, and Trichoptera) have been found to be important indicators of physical and chemical factors affecting aquatic systems [3, 35, 46]. In the present study, alpha diversity metrics for a similar limited subset of benthic taxa (i.e., Ephemeroptera, Trichoptera, and Odonata) show high consistency regardless of the taxonomic level employed (Table 1). The pattern of intersample ETO diversity was consistent between taxonomic levels, albeit with a wide range of reported values—3.13 morphological taxa to 7.63 genera per sample and 0.86% to 37.63% of total observations. The notable exception is order richness. This is understandable due to the narrow range of values possible–zero to three. The use of an HTS barcoding approach preserves the ability to easily focus on indicator groups as well as total biodiversity.

## Conclusion

The added taxonomic resolution supplied by HTS methods provides increased resolution of alpha, beta, and gamma biodiversity metrics. Critically, this information gain comes at a reduced cost, yet also provides increased discrimination between samples, sites, and river systems over more traditional methods.

The use of multiple biodiversity metrics in concert and the development of a multimetric index (MMI) has been proposed [47]. In practice, different suites of metrics have been found to be informative in environments subjected to differing stressors [41]. The use of HTS sequence data allows for the rapid generation of numerous biodiversity indices, to be investigated and combined in any number of MMI frameworks. While the CABIN protocol was employed here, the current method is also adaptable to any of the many benthic sampling protocols employed worldwide [6]. By utilizing the power of HTS, an increase in spatio-temporal sampling effort, combined with increased efficiency and data resolution is possible. Improved biomonitoring will empower biologists and managers to detect and respond to stressors threatening ecosystems more rapidly, yielding new insights into large-scale spatial patterning of planetary biodiversity.

## Supporting Information

S1 File Table A, Collection and locality data for all samples included in analysis. Table B, Additional biodiversity metrics calculated via four methods. (DOCX)
