# Supplementary material for: Large-Scale Biomonitoring of Remote and Threatened Ecosystems via High-Throughput Sequencing
Source: PLoS One. 2015 Oct 21;10(10):e0138432. doi: 10.1371/journal.pone.0138432 (PMC4619546; doi:10.1371/journal.pone.0138432)
Supplement: S1 File — (DOCX) [file pone.0138432.s001.docx]

**Supporting information and appendices**

**Table A. Collection and locality data for all samples included in analysis.**

| **River** | **Site** | **Latitude** | **Longitude** | **Date sampled** |
| --- | --- | --- | --- | --- |
| Athabasca | PAD1 | 58.60273˚N | 111.52612˚W | June 6, 2012 |
|  | PAD3 | 58.56475˚N | 111.51079˚W | June 6, 2012 |
|  | PAD4 | 58.50773˚N | 111.51802˚W | June 6, 2012 |
|  | PAD11 | 58.6384˚N | 111.59653˚W | June 6, 2012 |
| Peace | PAD14 | 58.87465˚N | 111.32484˚W | June 4, 2012 |
|  | PAD33 | 58.88236˚N | 111.39922˚W | June 5, 2012 |
|  | PAD37 | 58.83234˚N | 111.28074˚W | June 4, 2012 |
|  | PAD38 | 58.86389˚N | 111.58159˚W | June 5, 2012 |

**Table B. Additional biodiversity metrics calculated via four methods.**

|  | CABIN | DNA-Order | DNA-Family | DNA-Genus |
| --- | --- | --- | --- | --- |
| Welch 2-sample t-test of Simpson Index for Athabasca sites vs. Peace sites | t = -1.30, df = 17.10, p = 0.212 | t = -0.31, df = 21.96, p = 0.763 | t = -0.58, df = 21.99, p = 0.569 | t = 3.12, df = 15.90, p = 0.007 |
| Welch 2-sample t-test of Pielou’s Evenness for Athabasca sites vs. Peace sites | t = -0.31, df = 20.72, p = 0.761 | t = 0.26, df = 21.99, p = 0.797 | t = 0.12, df = 21.88, p = 0.907 | t = 3.29, df = 21.97, p = 0.003 |
| Permutational ANOVA (Bray-Curtis; raw sequences; 999 permutations) | Wetlands (F = 6.998, df = 1, p < 0.001); Sites (F = 5.393, df = 6, p < 0.001) | Wetlands (F = 12.496, df = 1, p < 0.001); Sites (F = 3.334, df = 6, p < 0.001) | Wetlands (F = 13.238, df = 1, p < 0.001); Sites (F = 3.692, df = 6, p < 0.001) | Wetlands (F = 11.827, df = 1, p < 0.001); Sites (F = 4.245, df = 6, p < 0.001) |
| Permutational ANOVA (Bray-Curtis; proportions; 999 permutations) | Wetlands (F = 5.551, df = 1, p = 0.003); Sites (F = 5.523, df = 6, p < 0.001) | Wetlands (F = 18.273, df = 1, p < 0.001); Sites (F = 4.098, df = 6, p < 0.001) | Wetlands (F = 19.722, df = 1, p < 0.001); Sites (F = 4.253, df = 6, p < 0.001) | Wetlands (F = 13.607, df = 1, p < 0.001); Sites (F = 4.471, df = 6, p < 0.001) |
| Permutational ANOVA (Sørensen; 999 permutations) | Wetlands (F = 11.642, df = 1, p < 0.001); Sites (F = 3.435, df = 6, p < 0.001) | Wetlands (F = 4.705, df = 1, p < 0.001); Sites (F = 2.574, df = 6, p < 0.001) | Wetlands (F = 6.037, df = 1, p < 0.001); Sites (F = 2.907, df = 6, p < 0.001) | Wetlands (F = 7.404, df = 1, p < 0.001); Sites (F = 3.036, df = 6, p < 0.001) |
| Homogeneity of multivariate dispersions, average distance to centroid – Bray-Curtis (raw sequences) | Athabasca - 0.35; Peace – 0.32; F = 0.726; p = 0.403 | Athabasca - 0.297; Peace – 0.366; F = 3.013; p = 0.097 | Athabasca - 0.317; Peace – 0.406; F = 4.549; p = 0.044 | Athabasca - 0.474; Peace – 0.443; F = 0.579; p = 0.455 |
| Homogeneity of multivariate dispersions, average distance to centroid – Bray-Curtis (proportions) | Athabasca - 0.318; Peace – 0.267; F = 2.404; p = 0.135 | Athabasca - 0.265; Peace – 0.340; F = 5.017; p = 0.036 | Athabasca - 0.277; Peace – 0.377; F = 7.677; p = 0.011 | Athabasca - 0.462; Peace – 0.423; F = 0.888; p = 0.356 |
| Homogeneity of multivariate dispersions, average distance to centroid – Sørensen | Athabasca - 0.20; Peace – 0.22; F = 0.499; p = 0.487 | Athabasca - 0.16; Peace – 0.13; F = 0.582; p = 0.454 | Athabasca - 0.23; Peace – 0.22; F = 0.252; p = 0.621 | Athabasca - 0.28; Peace – 0.27; F = 0.220; p = 0.644 |
